# The role of human umbilical cord mesenchymal stem cells-derived exosomal microRNA-431-5p in survival and prognosis of colorectal cancer patients

**DOI:** 10.1093/mutage/geac007

**Published:** 2022-04-23

**Authors:** Muwen Qu, Junyi Li, Zifu Hong, Fei Jia, Yinghua He, Lingling Yuan

**Affiliations:** 1 Anorectal Department of Guang’anmen Hospital of Chinese Academy of Chinese Medical Sciences, No. 5, Beixiange, Xicheng District, 100053 Beijing, China; 2 Surgical Department of Guang’anmen Hospital of Chinese Academy of Chinese Medical Sciences, No. 5, Beixiange, Xicheng District, 100053 Beijing, China; 3 Department of Dermatology, Dongzhimen Hospital, Beijing University of Traditional Chinese Medicine, No. 5, Haiyuncang, Dongcheng District, 100700 Beijing, China

**Keywords:** colorectal cancer, human umbilical cord mesenchymal stem cells-derived exosome, microRNA-431-5p, peroxiredoxin 1, survival, prognosis

## Abstract

We aim to discuss the role of miR-431-5p in colorectal cancer (CRC) progression via regulating peroxiredoxin 1 (PRDX1). miR-431-5p and PRDX1 expression were detected in CRC tissues and cells, and the relationship between miR-431-5p expression and prognosis of CRC patients was analyzed. Exosomes were extracted from human umbilical cord mesenchymal stem cells (hUCMSCs) and co-cultured with LoVo cells. MTT assay, flow cytometry and Transwell assay were implemented to test cell viability, apoptosis and invasion and migration ability, respectively. The tumor growth was determined as well, and the binding relation between miR-431-5p and PRDX1 was confirmed. miR-431-5p was downregulated and PRDX1 was upregulated in CRC, and miR-431-5p downregulation was associated with poor prognosis. hUCMSC-Exos suppressed the malignant behaviors of LoVo cells, and overexpression of miR-431-5p further aggravated the inhibitory effect of hUCMSC-Exos on LoVo cells. hUCMSC-Exos inhibited PRDX1 expression via miR-431-5p. PRDX1 was targeted by miR-431-5p. miR-431-5p serves as a prognostic biomarker in CRC, and hUCMSC-Exos transfer of miR-431-5p decelerates CRC cell growth by inhibiting PRDX1.

## Introduction

Colorectal cancer (CRC) is a common cause of cancer-associated death [1]. The underlying mechanisms of CRC are multifactorial. Risk factors include age, gender and lifestyle, and potential genetic factors that have little influence [2]. Despite improvements in screening, addressability, and awareness, a quarter of cases are still diagnosed with advanced CRC [3]. Multi-model treatment strategies could improve the overall survival (OS), but severe side effects such as weight loss accompany [4]. Thus, novel targets are urgently needed for CRC treatment.

Exosomes are extracellular vesicles [5] that are involved in multiple physiological and pathological processes [6]. Human umbilical cord mesenchymal stem cells (hUCMSCs) have great advantages of readily available source, self-renewal property, and immunomodulation [7]. It has been identified that exosomes contain microRNAs (miRNAs), mRNAs, DNA, and proteins [8]. miRNAs can suppress gene expression by mRNA degradation and translational suppression [9]. It is known that miR-4319 [10], miR-107 [11], and miR-431 could affect CRC progression [12], while the role of miR-431-5p remains largely unknown. Peroxiredoxins (PRDXs) are essential antioxidant proteins constituting the potent defense system to sustain redox balance by converting hydrogen peroxide to water. PRDX1 is a 23-kDa stress-triggered macrophage redox protein [13] that has been addressed to be related to tumor metastasis, angiogenesis [14], and inflammation in CRC [15]. We aim to identify the role of miR-431-5p conveyed by hUCMSC-Exos in the progression of CRC via regulating PRDX1, and inferred that hUCMSC-Exos may upregulate miR-431-5p to restrict the CRC cell growth via inhibiting PRDX1.

## Materials and methods

### Ethics statement

Written informed consent was acquired from all patients before this study. The protocol of this study was confirmed by the Ethics Committee of the Dongzhimen Hospital, Beijing University of Traditional Chinese Medicine. Animal experiments were strictly conformed with the Guide to the Management and Use of Laboratory Animals issued by the National Institutes of Health.

### Study subjects

CRC patients (*n* = 101) accepted resection in the Dongzhimen Hospital, Beijing University of Traditional Chinese Medicine were selected. All the patients were pathologically confirmed as primary adenocarcinoma and have not been treated with radio- or chemotherapy. Tumor and adjacent normal tissues were resected, frozen, and preserved at −80°C. OS was calculated from the resection to death time or the last follow-up visit.

### Cell culture, transfection and treatment

Human CRC cell lines (Caco-2, SW480, SW620, LoVo, and HCT 116) and normal epithelial cells NCM460 were obtained from BeNa Culture Collection (Beijing, China) and cultured in specific medium. hUCMSCs was acquired from BeNa Culture Collection and cultured in Dulbecco’s Modified Eagle Medium (DMEM) with 10% fetal bovine serum (FBS) and 1% penicillinstreptomycin (P/S).

The hUCMSCs were transfected with mimic negative control (NC), miR-431-5p mimic , inhibitor NC, and miR-431-5p inhibitor. The corresponding exosomes extracted from the transfected hUCMSCs were named: NC-mimic-exo, miR-431-5p-mimic-exo, NC-inhibitor-exo and miR-431-5p-inhibitor-exo. LoVo cells were also transfected with inhibitor NC, miR-431-5p inhibitor and miR-431-5p inhibitor + PRDX1 siRNA. The mimic, miR-431-5p-mimic, NC-inhibitor, miR-431-5p-inhibitor, and si-PRDX1 were provided by GenePharma (Shanghai, China). Cells were cultured in six-well plates (5 × 10^5^ cells/well). Cell transfection was performed via the Lipofectamine 2000 reagent (Invitrogen, New York, California, USA). Cells with 48-h transfection were collected for subsequent experiments. To observe the effect of hUCMSCs-derived exosomes on CRC cells, 100 μg of the corresponding hUCMSCs-derived exosomes (NC-mimic-exo, miR-431-5p-mimic-exo, NC-inhibitor-exo and miR-431-5p -inhibitor-exo) were co-cultured with LoVo cells for 48 h, and cells were harvested for follow-up experiments.

### Isolation and identification of hUCMSCs

hUCMSCs immunophenotypes were evaluated. Harvested cells were labeled using mouse anti-human monoclonal antibodies: fluorescein isothiocyanate (FITC)-conjugated CD105 (20 μl/10^6^ cells), CD90 (5 μl/10^6^ cells), CD73 (5 μl/10^6^ cells), CD14 (20 μl/10^6^ cells), CD34 (10 μl/10^6^ cells), CD45 (10 μl/10^6^ cells), and HLA-DR (1 μl/10^6^ cells) (Abcam Inc., MA, USA). FITC-labeled mouse anti-human immunoglobulin G1 (IgG1) was used as the homotype control. MSC phenotypes were identified using a flow cytometer (BD Biosciences, NJ, USA).

### Isolation and identification of hUCMSC-Exos

To eliminate interference from FBS-derived exosomes in the cell culture medium, the FBS was centrifuged at 100,000 × *g* for 18 h. The culture medium was replaced with medium containing 10% exosome-free FBS. After 48 h of incubation, the cell supernatant was collected. The collected supernatant was centrifuged at 500 *g* for 15 min at 4°C to remove cell debris, and followed by centrifugation at 2000 to remove apoptotic vesicles and centrifugation at 10 000 *g* for 20 min at 4°C to remove large vesicles. The supernatant was then filtered by a 0.22-μm filter, centrifuged at 110 000 *g* for 70 min at 4°C, resuspended with PBS, and then centrifuged in the same condition. The downstream experiments were performed by further superseding with 100 μl sterile PBS. The morphology of exosomes was observed by a transmission electron microscope (TEM). The exosomes were diluted and dropped onto the copper mesh for staining with 3% sodium phosphotungstate solution (pH = 6.8). The particle size of exosomes was measured by Zetasizer Nano ZS90 (Malvern Instrument, Malvern, USA). Western blot analysis was used to detect CD9 (1:1000), CD81 (1:1000), and TSG101 (1:1000; Abcam).

### Uptake of hUCMSC-Exos

Exosomes and PKH26 were incubated for 30 min and centrifuged at 4°C for 60 min. After incubation with LoVo cells, PKH26-labeled exosomes were stained with 4ʹ,6-diamidino-2-phenylindole 2 and viewed under a IX53 fluorescent microscope (Olympus, Tokyo, Japan).

To explore the transfer of miR-431-5p, FITC-miR-431-5p was packaged into exosomes using electroporation, and isolated from 300 μl hUCMSCs cell culture-conditioned medium and packaged with FITC-miR-431-5p. Briefly, 30 μl FITC-miR-431-5p was added to 200 ng exosomes in 400 μl electroporation buffer (21% Optiprep, 25 mm KCl, 100 mm potassium phosphate, pH 7.2) and transferred to a 4-mm electroporation tube (Eppendorf, Hauppauge, NY, USA). Samples were electroporated in an E2510 electroporator (Eppendorf, Hauppauge, NY, USA) with three pulses at 0–2000 V and stored at 4°C for 5 min. The exosomes containing FITC-miR-431-5p were labeled with Dil (red), and finally incubated with LoVo cells for 48 h. The co-localization of FITC and Dil was observed in recipient cells using a fluorescence microscope (ECLIPSE E800, Nikon, Japan).

### Reverse transcription-quantitative polymerase chain reaction

Total RNA in tissues, cells, and exosomes was extracted with Trizol reagent (Sigma-Aldrich, MO, USA). For miRNA, miRcute miRNA First-Strand cDNA Synthesis kit (Tiangen Biotech, Beijing, China) was used for RNA reverse transcription. For mRNA, PrimeScript™ RT reagent (TaKaRa) used to synthesize cDNA. the TB Green™ Premix Ex Taq™ II kit (TaKaRa) was utilized to perform qRT‐PCR on a Real‐Time PCR Detection System (Bio‐Rad). The results were normalized with glyceraldehyde phosphate dehydrogenase (GAPDH) and U6. Primer sequences were shown in Supplementary Table 1.

### 3-(4,5-Dimethyl-2-thiazolyl)-2,5-diphenyl-2-H-tetrazolium bromide assay

LoVo cells were stained, respectively, on the first, second, third, and fourth day of culture. Each well was incubated with 100 μl 3-(4,5-dimethyl-2-thiazolyl)-2,5-diphenyl-2-H-tetrazolium bromide (MTT) solution (0.5 mg/ml, Sigma) for 4 h and supplemented with 150 μl dimethyl sulfoxide for 10 min. Optical density at 490 nm was analyzed by a microplate reader (Tecan, Switzerland).

### Transwell assay

Migration assay: 0.2 ml cells (concentration: 2 × 10^5^/ml) were seeded into Transwell (Corning, NY, USA) apical chambers and incubated with 100 μl serum-free medium. The basolateral chambers were added with medium containing 20% FBS and incubated for 16 h. The migrated cells were fixed with 4% paraformaldehyde and stained with 0.1% crystal violet.

Invasion assay: apical chambers were coated with Matrigel (BD Biosciences) and other steps were the same as migration assay.

### Flow cytometry

Apoptosis of LoVo cells was determined by flow cytometry as previously described [16] and the results were analyzed by a flow cytometer (BD Biosciences).

### Dual luciferase reporter gene assay

PRDX1 sequences containing wild-type (WT-Type) or mutated (MUT-Type) miR-431-5p binding sites were synthesized and inserted into pmiR-GLO luciferase reporter plasmid (Promega, WI, USA), respectively. LoVo cells were transfected using Lipofectamine 2000 (Invitrogen). PRDX1-WT or PRDX1-MUT were co-transfected with miR-431-5p-mimic or NC-mimic, respectively. After 24-h transfection, the luciferase activity was detected by the Glomax20/20 luminometer (Promega).

### Western blot analysis

Proteins were performed with gel electrophoresis and transferred onto membranes, which were blocked with 5% skim milk powder and incubated with primary antibodies PRDX1 (1:1000) and GAPDH (1:1000; Abcam) and with relative secondary antibody. After development by enhanced chemiluminescent reagent, bands were exposed on the Image Quant LAS 4000C gel imager (General Electric Company, USA).

### Subcutaneous tumorigenesis in nude mice

Twenty-four nude mice (aged 4–6 weeks) were randomly assigned into 4 groups (6 mice per group). LoVo cells (1 × 10^6^ cells/mouse) were injected subcutaneously into the flank of nude mice. Meanwhile, a dosage of 5 mg exosomes administered into mice via tail vein injection once every 3 days for 2 weeks. Tumor size was measured with calipers after the tumor cell injection once every 7 days for a total period of 4 weeks. The tumor volume = length × width^2^ × 0.5. Mice were euthanized on the 28th day of the tumor cell injection and the xenografts were isolated and weighed.

### Statistical analysis

All data analyses were conducted using SPSS 21.0 software (IBM, NY, USA). The measurement data were expressed as mean ± standard deviation. The *t*-test was performed for comparisons between two groups, analysis of variance (ANOVA) was used for comparisons among multiple groups and Tukey’s *post hoc* test was used for pairwise comparisons after ANOVA. Chi square test was carried out to analyze the relationship of miR-431-5p expression clinicopathological characteristics of CRC patients. Kaplan–Meier method was used to analyze the survival and Pearson test was employed to assess the correlation between miR-431-5p and PRDX1. *P-*value < 0.05 was indicative of statistically significant difference.

## Results

### miR-431-5p is downregulated in CRC and is related to the poor prognosis of CRC patients

**Figure 1.**
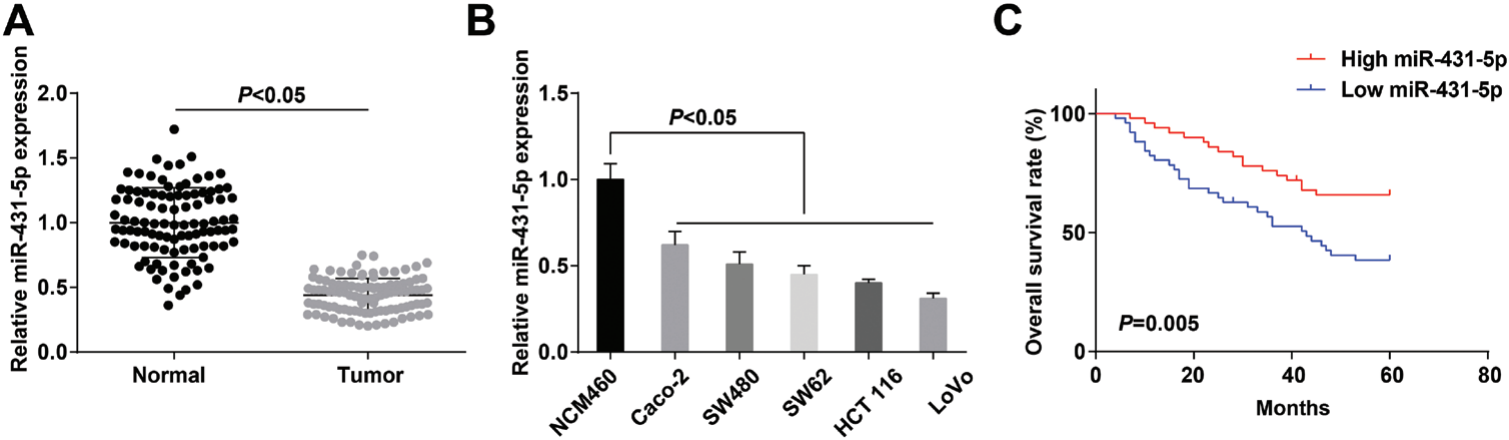
miR-431-5p is downregulated in CRC, and is related to poor prognosis of CRC patients. (A) Expression of miR-431-5p in CRC tissues and adjacent normal tissues; (B) expression of miR-431-5p in CRC cells and normal epithelial cells; (C) the predictive role of miR-431 in CRC patient prognosis was analyzed by Kaplan–Meier method.

miR-431-5p downregulation has been previously identified in cancers [12, 17]. To assess the role of miR-431-5p in CRC, its expression in tissues was determined. We found that CRC tissues had lower miR-431-5p expression than adjacent normal tissues (Fig. 1A). miR-431-5p expression in cells was also evaluated and it was discovered that miR-431-5p was downregulated in five CRC cell lines. The most significant downregulation of miR-431-5p expression was observed in LoVo cells, which were thereby selected for follow-up experiments (Fig. 1B).

CRC patients were separated into high and low miR-431-5p expression groups based on the median miR-431-5p expression to assess the relation between miR-431-5p expression and clinicopathological characteristics of CRC patients (*n* = 51). The results indicated that low miR-431-5p expression was related to tumor-node-metastasis (TNM) stage, lymph node metastasis (LNM), and differentiation degree (Supplementary Table 2). Moreover, we found from Kaplan–Meier method that patients with low miR-431-5p expression had a shorter OS (Fig. 1C).

### hUCMSC-Exos suppress CRC cell growth

**Figure 2.**
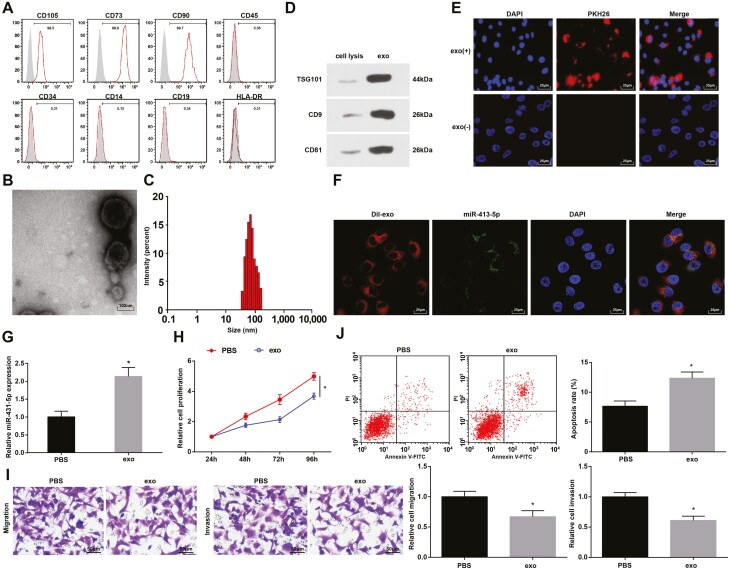
hUCMSC-Exos suppress CRC cell growth. (A) Surface markers of hUCMSC-Exos were determined by flow cytometry; (B) hUCMSC-Exos were observed under a TEM; (C) particle diameter distribution of hUCMSC-Exos was analyzed by Zetasizer Nano ZS90; (D) protein expression of CD9, CD81, and TSG101 in hUCMSC-Exos was determined by western blot analysis; (E) uptake of hUCMSC-Exos by LoVo cells was observed under a fluorescent microscope; (F) the labeled fluorescent FITC-miR-431-5p and exosomes co-localization in LoVo cells was observed by the immunofluorescence microscopy, FITC-miR-431-5p labeled exosomes in green, 4ʹ,6-diamidino-2-phenylindole-stained nuclei were in blue; Dil-labeled exosomes were in red; (G) miR-431-5p expression in LoVo cells; (H) proliferation of LoVo cells was determined by MTT assay; (I) migration and invasion of LoVo cells were determined by Transwell assay; (J) apoptosis of LoVo cells was assessed using flow cytometry; *P < 0.05 versus the PBS group.

The surface markers of hUCMSCs were determined and we found that CD90 and CD105 were positive while CD14, CD19, and CD34 were negative (Fig. 2A). It was observed under a TEM that hUCMSC-Exos were spherical or ellipsoidal vesicles with complete envelope and similar morphology (Fig. 2B). It was observed by the Zetasizer Nano ZS90 that the particle diameter ranged from 30 to 120 nm  (Fig. 2C). Moreover, the protein expression of CD9, CD81, and TSG101 was higher in hUCMSC-Exos than in hUCMSCs (Fig. 2D).

PKH26-labeled exosomes were co-cultured with LoVo cells and it was observed under a fluorescent microscope that green fluorescence appeared in the cytoplasm, suggesting the successful uptake of exosomes by LoVo cells  (Fig. 2E). To further explore the transfer of miR-431-5p, we electrotransferred FITC-miR-431-5p (green) into exosomes from hUCMSCs, added Dil tags (red), and incubated LoVo cells for 48 h. Co-localization of FITC and Dil was observed in the receptor LoVo cells, indicating that the FITC-miR-431-5p-containing cellular exosomes were internalized by LoVo cells (Fig. 2F). miR-431-5p expression in LoVo cells was assessed and we found that miR-431-5p was upregulated with hUCMSC-Exos treatment (Fig. 2G). It was found in MTT assay, Transwell assay, and flow cytometry that hUCMSC-Exos suppressed proliferation, migration, and invasion of LoVo cells; results of flow cytometry implied that the exosomes promoted LoVo cell apoptosis (Fig. 2H–J).

### hUCMSC-Exos overexpressing miR-431-5p represses CRC cell growth

**Figure 3.**
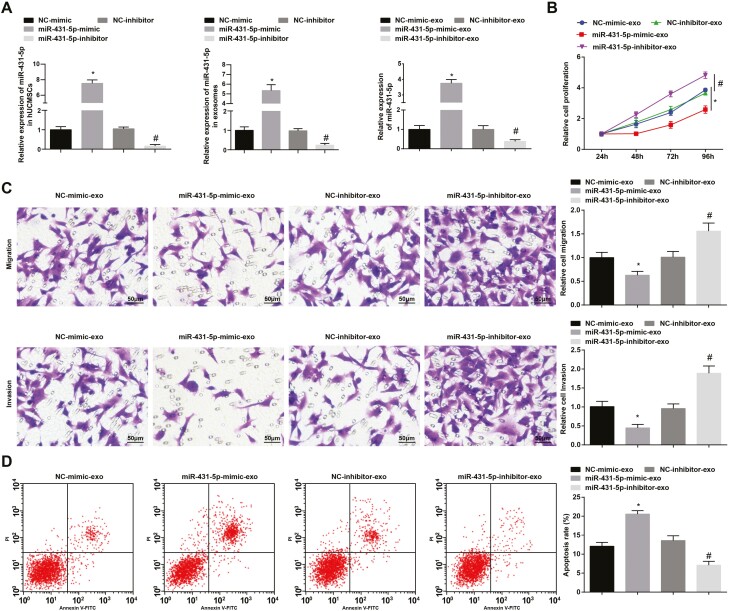
hUCMSC-Exos overexpressing miR-431-5p represses CRC cell growth. (A) miR-431-5p expression in hUCMSCs, hUCMSC-Exos, and LoVo cells; (B) proliferation of LoVo cells was determined by MTT assay; (C) migration and invasion of LoVo cells were determined by Transwell assay; (D) apoptosis of LoVo cells was assessed using flow cytometry; *P < 0.05 versus the NC-mimic-exo group, ^#^*P* < 0.05 versus the NC-inhibitor-exo group.

miR-431-5p was altered in hUCMSC-Exos to assess its role in CRC cells. Outcomes of reverse transcription-quantitative PCR indicated that miR-431-5p-mimic upregulated miR-431-5p and miR-431-5p-inhibitor downregulated miR-431-5p in hUCMSCs, hUCMSC-Exos, and co-cultured LoVo cells (Fig. 3A).

Phenotypes of LoVo cells were determined: hUCMSC-Exos overexpressing miR-431-5p restricted malignant behaviors of LoVo cells than NC-mimic-exo treatment, whereas hUCMSC-Exos inhibiting miR-431-5p induced malignant behaviors of LoVo cells than NC-inhibitor-exo treatment (Fig. 3B–D).

### miR-431-5p targets PRDX1

**Figure 4.**
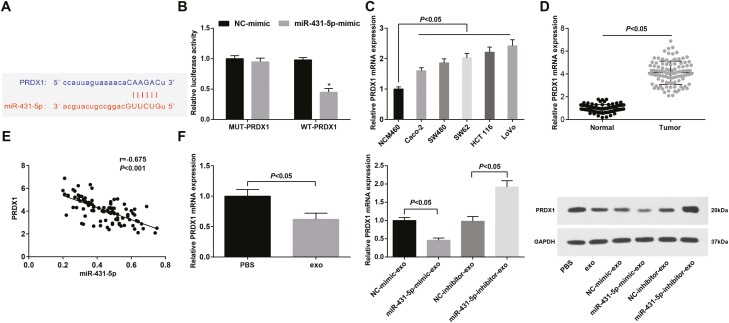
miR-431-5p targets PRDX1. (A) Binding site between miR-431-5p and PRDX1 was predicted at Starbase; (B) binding relation between miR-431-5p and PRDX1 was confirmed by dual luciferase reporter gene assay; (C) PRDX1 expression in CRC cell lines and normal epithelial cells; (D) PRDX1 expression in CRC tissues and adjacent normal tissues; (E) correlation between expression of miR-431-5p and PRDX1 was analyzed by Pearson test; (F) expression of PRDX1 in LoVo cells; *P < 0.05 versus the NC-mimic group.

PRDX1 upregulation has been previously identified in CRC [14, 18]. It was predicted at Starbase that there existed binding sites between miR-431-5p and PRDX1 (Fig. 4A). Moreover, it was further confirmed that the co-transfection of WT-PRDX1 and miR-431-5p-mimic reduced the luciferase activity (Fig. 4B). PRDX1 expression in CRC was evaluated and the results suggested that CRC cell lines displayed high PRDX1 expression (Fig. 4C); CRC tissues also exhibited elevated PRDX1 expression (Fig. 4D). Pearson test indicated that miR-431-5p expression was negatively related to PRDX1 expression in CRC tissues (Fig. 4E). After co-culture, we found that hUCMSC-Exos downregulated PRDX1 in LoVo cells, and hUCMSC-Exos overexpressing miR-431-5p repressed PRDX1 expression compared with NC-mimic-exo treatment; while hUCMSC-Exos inhibiting miR-431-5p upregulated PRDX1 in comparison to NC-inhibitor-exo treatment (Fig. 4F).

### PRDX1 downregulation reverses the effects of miR-431-3p downregulation on LoVo cells

**Figure 5.**
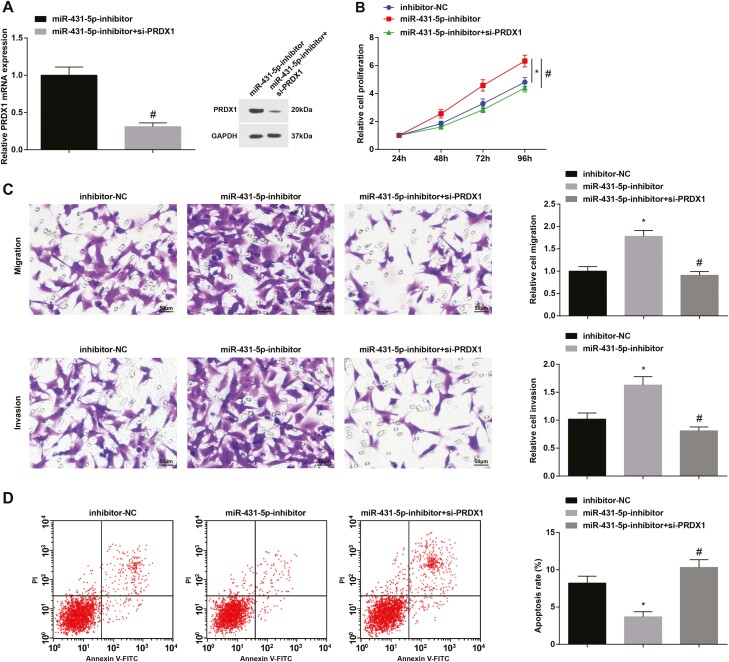
PRDX1 downregulation reverses the role of miR-431-3p downregulation for LoVo cells. (A) Expression of PRDX1 in LoVo cells; (B) proliferation of LoVo cells was determined by MTT assay; (C) migration and invasion of LoVo cells were determined by Transwell assay; (D) apoptosis of LoVo cells was assessed using flow cytometry; ^*^P < 0.05 versus the inhibitor-NC group; ^*^P < 0.05 versus the miR-431-5p inhibitor group.

PRDX1 expression in LoVo cells was assessed and we observed that compared with the miR-431-5p inhibitor group, PRDX1 expression was decreased in the miR-431-5p inhibitor + si-PRDX1 group (Fig. 5A). Biological functions of LoVo cells were determined and it came out that si-PRDX1 abolished miR-431-3p inhibitor-induced effects on LoVo cells (Fig. 5B–D).

### hUCMSC-Exos overexpressing miR-431-5p inhibits tumor growth

**Figure 6.**
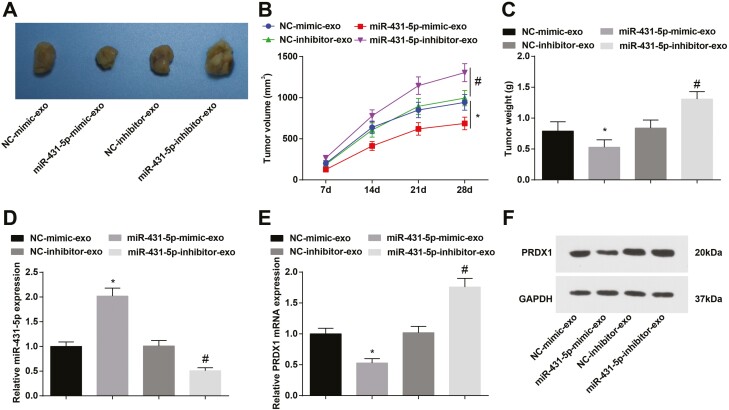
hUCMSC-Exos overexpressing miR-431-5p represses tumor growth. (A) Tumors from the nude mice; (B) tumor volume of nude mice; (C) tumor weight of nude mice; (D) miR-431-5p expression in the xenografts; (E, F) PRDX1 expression in the xenografts; *P < 0.05 versus the NC-mimic-exo group, ^#^*P* < 0.05 versus the NC-inhibitor-exo group.

Results of our *in vivo* experiments indicated that hUCMSC-Exos overexpressing miR-431-5p repressed weight and volume of the xenografts compared with NC-mimic-exo treatment; while hUCMSC-Exos inhibiting miR-431-5p increased the xenograft weight and volume in comparison to NC-inhibitor-exo treatment (Fig. 6A–C). Meanwhile, miR-431-5p was found to downregulate PRDX1 in tumors (Fig. 6D–F). These findings evidenced that hUCMSC-Exos overexpressing miR-431-5p could decelerate the tumor development.

## Discussion

CRC has a higher incidence and mortality in developed countries [19]. We found that hUCMSC-Exos upregulated miR-431-5p to restrict CRC cell growth via inhibiting PRDX1.

LoVo cells were treated with HUCMSC-Exos to clarify their effects on GC cell growth, and we found that hUCMSC-Exos were able to suppress the growth of CRC cells. Similarly, it has been discovered that hUCMSC-Exos reduced pancreatic ductal adenocarcinoma cell growth [20] and restrained malignant behaviors of breast cancer cells [21]. It has been described that extracellular vesicles derived from hUCMSCs suppressed colon adenocarcinoma cell proliferation and migration [22] and MSCs-derived exosomes inhibited angiogenesis, proliferation, and migration of breast cancer cells [23]. Moreover, this study suggested that hUCMSC-Exos could upregulate miR-431-5p expression and downregulate PRDX1 expression.

We determined miR-431-5p expression in CRC tissues and cells, and we found that miR-431-5p was downregulated in CRC. miR-431-5p has once been found to be downregulated in CRC cancer tissues and cells [24], and miR-431 was also downregulated in CRC [25]. Considering the dysregulation of miR-431-5p in CRC, we analyzed the predictive role of miR-431-5p in CRC patients, and our results implied that low miR-431-5p expression suggested a poor prognosis, and miR-431-5p expression was associated with TNM, LNM, and differentiation of CRC patients. Wu *et al*. have illuminated that miR-431-5p was associated with the OS of esophageal carcinoma patients [26], and miR-431 was related to LNM and TNM stage of patients with hepatocellular carcinoma [27]. Results of our experiments indicated that overexpressed miR-431-5p conveyed by hUCMSC-Exos repressed the development  of CRC cells while inhibited miR-431-5p had opposite effects on CRC cells. Similarly, it has been recently illustrated that the ectopic expression of miR-431-5p repressed the proliferation and invasion of CRC cells [24], and Su *et al*. have validated that the migration of CRC cells transfected with miR-431 mimic was markedly reduced, while cells transfected with miR-431 inhibitor performed reverse alterations [25].

It is known that miRNAs can post-transcriptionally modulate target gene expression [28]. Our study suggested that PRDX1, targeted by miR-431-5p, was upregulated in CRC tissues and cells. Similarly, studies have uncovered that PRDX1 exhibited a high expression level in CRC [14, 15]. However, the binding relationship between miR-431-5p and PRDX1 remains scarcely discussed. Furthermore, we discovered that PRDX1 downregulation mitigated the impacts of downregulated miR-431-5p on CRC cells. It has been elucidated that PRDX1 inhibition could suppress the growth of CRC cells [18].

In conclusion, we found that miR-431-5p served as a predictive role in CRC patients’ prognosis, and upregulated miR-431-5p conveyed by hUCMSC-Exos suppresses CRC progression by inhibiting PRDX1. Our study may contribute to CRC treatment, while there remains much to do to reveal the underlying mechanisms.

## Supplementary Material

geac007_suppl_Supplementary_Tables
