# Supplementary material for: The role of human umbilical cord mesenchymal stem cells-derived exosomal microRNA-431-5p in survival and prognosis of colorectal cancer patients
Source: Mutagenesis. 2022 Apr 23;37(2):164–71. doi: 10.1093/mutage/geac007 (PMC9071100; doi:10.1093/mutage/geac007)
Supplement: geac007_suppl_Supplementary_Tables [file geac007_suppl_supplementary_tables.docx]

**Supplementary Table 1** Primer sequences for RT-qPCR

| Gene | Primer sequence (5’-3’) |
| --- | --- |
| miR-431-5p | F: TGTCTTGCAGGCCGTCATGCA |
|  | R: Universal primer |
| PRDX1 | F: CCATAAACGACCTTCCTGTT |
|  | R: GAGAAATATTCTTTGCTCTTCTGGAC |
| U6 | F: ATTGGAACGATACAGAGAAGATT |
|  | R: GGAACGCTTCACGAATTTG |
| GAPDH | F: ATGCTGCCCTTACCCCGG |
|  | R: TTACTCCTTGGAGGCCATGTAGG |

Note: F, forward; R, reverse; miR-431-5p, microRNA-431-5p; PRDX1, peroxiredoxin 1; GAPDH, glyceraldehyde phosphate dehydrogenase.

**Supplementary Table 2** Relationship between miR-431-5p expression and clinicopathological characteristics of CRC patients

| Clinicopathological  characteristics | n | miR-431-5p expression | | *P* |
| --- | --- | --- | --- | --- |
|  |  | High expression  (n = 50) | Low expression  (n = 51) |  |
| Age (year) |  |  |  |  |
| ≥ 59 | 45 | 21 | 24 | 0.690 |
| < 59 | 56 | 29 | 27 |  |
| Gender |  |  |  |  |
| Male | 53 | 27 | 26 | 0.843 |
| Female | 48 | 23 | 25 |  |
| Tumor diameter |  |  |  |  |
| < 5 cm | 44 | 20 | 24 | 0.549 |
| ≥ 5 cm | 57 | 30 | 27 |  |
| LNM |  |  |  |  |
| Yes | 59 | 23 | 36 | 0.016 |
| No | 42 | 27 | 15 |  |
| TNM stage |  |  |  |  |
| I-II | 37 | 26 | 11 | 0.002 |
| III | 64 | 24 | 40 |  |
| Differentiation |  |  |  |  |
| Well or moderate | 76 | 43 | 33 | 0.020 |
| Poor | 25 | 7 | 18 |  |

Note: CRC, colorectal cancer; miR-431-5p, microRNA-431-5p; LNM, lymph node metastasis; TNM, tumor-node-metastasis. Data in the table were enumeration data and analyzed by chi-square test.
